# General Public Views, Attitudes, and Experiences toward Drug Safety in Dubai, United Arab Emirates: A Qualitative Approach

**DOI:** 10.3390/pharmacy7010019

**Published:** 2019-02-07

**Authors:** Doaa Alkhalidi, Shazia Qasim Jamshed, Ramadan Mohamed Elkalmi, Mirza Rafi Baig, Adeel Aslam, Mohamed Azmi Hassali

**Affiliations:** 1 Department of Pharmacy Practice, Kulliyyah of Pharmacy, IIUM, Kuantan, Pahang 25200, Malaysia; dr.doaakamal@dpc.edu (D.A.); adeelaslam224@gmail.com (A.A.); 2 Department of Pharmacy Practice, Faculty of Pharmacy, Universiti Teknologi Mara, Shah Alam, Selangor 42300, Malaysia; edriph@gmail.com; 3 Department of Clinical Pharmacy & Pharmacotherapeutics, Dubai Pharmacy College, Dubai, UAE; dr.mirzabaig@dpc.edu; 4 Discipline of Social and Administrative Pharmacy, School of Pharmaceutical Sciences, Universiti Sains Malaysia, Penang 11800, Malaysia; azmihassali@usm.my

**Keywords:** public, drug safety, ADR reporting, Dubai

## Abstract

Ensuring drug safety among the patients is the main domain of pharmacovigilance activities worldwide. A pharmacovigilance system was established in the United Arab Emirates (UAE) in 2008. Research evidence reflects that the current system is lacking in active participation from patients, and also, the inadequate role of healthcare professionals is anticipated. In this context, it is pertinent to know the general public’s understandings and their patterns of safe use of medication, which are unexplored areas in Dubai, UAE. The current study aimed to explore the public views, attitudes, and experiences toward medication safety, and to explore key factors enhancing the safe use of medications among the public in Dubai. This study adopted a qualitative approach and face-to-face, 14 in-depth interviews with public individuals, selected purposively using the snowball sampling technique. The interviews were conducted in different places in Dubai recorded and transcribed verbatim and thematically analyzed for data analysis. Reporting of adverse drug reaction was not well-known among all the participants. Public views towards safe use of medicines were limited to the side effects of the consumed medicines only, and to a lesser extent to the inappropriate indication and dosage. Most of the participants mentioned that gaining knowledge about the side effects of the prescribed drug was the main reason for reading the patient information leaflet. Quite a few participants have experienced side effects while consuming their medicines and they were unsure of how to deal with the situation. The current research also reflected the lack of proper communication between pharmacists and physicians in managing drug safety issues. Conclusively, the current research revealed gaps in public views regarding medication’s safety, which consequently may impact their attitudes during the course of medication use. Efforts need to be strengthened to enhance positive views and attitudes of the public towards medication safety and ADR reporting in the UAE.

## 1. Introduction

The knowledge and pattern of using drugs remain important issues in the field of medication safety [1]. Revolution of medication-related advertisements via internet and other social media is a common feature, evoking patient’s inquisitiveness and feeling about a number of choices for selection. [2]. This also makes the patient a major partner in treatment-making decisions [3]. Therefore, an individual’s awareness of their medications is necessary to ensure their safe use [3]. It is evident from the previous research that medication safety is more important than finding signals of adverse drug reactions [3], and it is more likely to prevent harmful effects on patients and promote rational and safe prescription practices [3]. The rational use of medicines is defined as when “patients receive the appropriate medicines, in doses that meet their requirements, for an adequate period, and at the lowest cost both to the patient and the community” [4]. Accordingly, the irrational use of medicines is critical to the extent of interfering with the patients’ medications and safety [5]. The accessibility of medicines does not ensure their appropriate use among individuals and consumers [5,6]. Therefore, optimizing the safe use of medicines among the general public is highly necessary in our societies to ensure medication safety and consequently to reduce the incidence of adverse drug reactions (ADRs). However, it is still a challengeable practice [7], and previous studies reported ADRs being significantly associated with high rates of morbidity and mortality [7,8].

Ensuring drug safety among patients remains a major domain of pharmacovigilance activities worldwide [1]. The pharmacovigilance system was established in the UAE in 2008. The literature has shown that the current system is lacking an active participation of patients in addition to an insufficient role of healthcare professionals [9,10]. The pharmacovigilance system in the UAE is a composite of different local pharmacovigilance centers in the UAE that are connected to the main pharmacovigilance unit in the UAE, linked to the Uppsala Monitoring Centre and the WHO. The ADR reporting form is available electronically on the websites of the Ministry of Health (MOH) and other healthcare regulatory authorities in the UAE. The applicability of the policy of reporting ADR involved all healthcare providers (private and government) and public in the UAE. Upon receiving the reported ADRs, the concerned expert committees’ members have the main responsibility for analysis and evaluation of ADR causing factors, issuing circulars and publications related to the ADRs [11]. 

A large body of literature cited consumers’ negative views of medications which generally interrupt achieving their therapeutic benefits [4,5,6]. Patients with low health literacy demonstrated negative attitudes towards their prescribed treatments and self-care plans and reported more treatment-related errors, thus making less use of preventive measures compared to patients having high health literacy. In 2014, research from Thailand concluded that public knowledge about medication safety should be strengthened to assure positive attitudes toward medication use [12], which is in line with many other studies [13,14,15,16]. Therefore, more efforts are advocated to have knowledgeable individuals towards medications with good capability of coping with the undesirable effects [17].

Currently, the MOH and the health sectors in the UAE are aiming at high standards of action to maintain medication safety up to the mark. Relevant conferences are conducted frequently for healthcare professionals, pharmacovigilance centers have been established, and adverse drug reactions (ADR) reporting system has been officially activated. Studies have described medication safety matters in the UAE among healthcare professionals; however, to the best of our knowledge no study is reported to describe the real situation among the lay public. The current research aimed to explore the public’s views, attitudes, and experiences toward medication safety, and to explore the key factors enhancing the safe use of medications in Dubai, UAE.

## 2. Methodology

A qualitative approach was adopted and a semi-structured interview guide (SSIG) was used as research instrument. Qualitative studies are implemented in order to explore in detail the individuals’ views, thoughts, behaviors, and experiences in particular settings where people can explain their priorities and concerns deeply [18]. Qualitative research provides a clear understanding about a particular type of problem that enables health and educational policies to be developed [19,20]. In the context of pharmacy practice, qualitative studies help the pharmacist in exploring the underlying causes that interfere with the patient care process [21].

The SSIG was developed upon referring to the relevant literature and by the guidance of qualitative studies which were conducted in the field of medications safety [5,14]. The SSIG had different sections inquiring about the participant’s knowledge about medicines in general, participant’s beliefs about medicine’s benefits and risks, any recent experiences taking prescriptions and/or over the counter (OTC) medicines or supplements, their reactions to new risks of existing medicines, and patient’s feedback or comments regarding the best source of drug-related information. The SSIG was studied and revised by two qualitative researchers/co-authors (RME and MAH). 

Ethical approval was obtained from the Ethical Research Committee of the Ministry of Health and Prevention (MOHAP) in the UAE (Approval Reference No: MOHAP/DXB/SUBC/No. 15/2017) prior to data collection.

### 2.1. Sampling and Recruitment

The sampling technique in the current research was purposive snowballing, in which the researcher explicitly selected the participants who would be able to contribute to the topic and willing to share their own experiences regarding medication safety. Qualitative researchers must select a sample from which most can be learned in order to understand from the perspectives of the participants, as people who can provide a description of experiences are the ones who generally enrich the research data [22]. In this research, the recruited participants were lay public who were selected on the criteria of each participant being 18 years and older, able to read, speak, and write Arabic and must not be related to the medical or healthcare field. It is pertinent to highlight that neither the interviewer nor any researcher/co-author has any personal or professional acquaintance with any of the interviewees. 

The study and its aims were explained to each participant and if found willing to participate, he/she was requested to provide the researcher with any means of contact to arrange the interviews in advance. Every participant was informed that the interview would be recorded, and thus, an informed consent document having details of the study and how its confidentiality would be maintained was submitted to him/her for a careful read and their approval signature before attending the interview. The final participants were called later to determine the suitable time and place to proceed with the interview process. The interviews were conducted in the agreed place with the informed consent submitted to the researcher. The participants were selected from both genders and exhibited both health and disease states equally to achieve broader views and perspectives on drug safety. 

### 2.2. Data Collection

The interviews were carried out from September–October 2017 in Dubai. In-depth face-to-face interviews were performed. Before starting the interview, the researcher had an ice-breaking session with every participant. Moreover, the researcher notified each participant once the recording started and assured him/her that confidentiality and anonymity would be strictly maintained. After, the researcher introduced the topic of the research to the interviewee and explained the importance of such studies for community safety. This came as an effort to build-up an approachable relationship between the two parties as a means of establishing flexibility. Establishing a trusting and open relationship with the participant before and during an interview is necessary in the qualitative aspect of a study [23]. All interviews were conducted by the researcher (first author) and all were tape- recorded. The approximate duration of the interviews was 45 minutes to one hour. Participants were interviewed till saturation achieved, and thus no new interviews were conducted after 14 interviews. The emergent data was reflective of having no new themes after the 12 interviews, but two more interviews were carried out to ensure saturation [24]. 

### 2.3. Data Analysis

Participants’ responses were analyzed using inductive thematic analysis approach whereas themes and subthemes were generated as per the next steps. The recorded interviews have been transcribed verbatim by the researcher so that each interview was converted to a full detailed written text as it is always recommended for the recorded data to be transcribed exactly (word for word) from the interview and not paraphrased followed by accuracy check in transcription [25]. The identities of the participants were replaced by a certain symbol (P1–P14), where the real identities were deleted from the transcripts to maintain their confidentiality. After that, all the full transcripts were read carefully twice in an attempt to correlate the participant’s responses with the field of the research deeply. Authors have suggested that the qualitative researchers should read between the lines to interpret the meaning of data in the context of the research and to truly understand the world from their perspectives [19]. Once the researcher completed the transcribing and checking of participants interviews, coding was performed [26]. Coding is defined as “identification of topics, issues, similarities, and differences that are revealed through the participants’ narratives and interpreted by the researcher” [26]. It was performed manually using the hard copies of the transcripts by two researchers (RME and SQJ) and labeling each section in the written texts. Manual coding is possible with small and straightforward data sets [21]. The resulted codes from previous step were drawn together and compared for similarities and differences. Accordingly, new categories have been developed where each category contained the cluster of relevant codes. The process is called “theming “as each theme provided meaningful title which indicated what the participants said from the research point of view [26] and subsequently sub-themes were produced.

## 3. Results

### 3.1. Demographic Data

Overall, 14 participants (P1–P14) aged between 22 and 64 years were included in the study. A total of 14% (*n* = 2) were locals (Emirati) and 86% (*n* = 12) were non-local (expatriates). About 43% (*n* = 6) of the participants were male and 57% (*n* = 8) were females. Their education levels ranged between secondary school and university qualifications. Seven participants mentioned that they had chronic diseases, one of them had acute coronary syndrome, one participant with hyperlipidemia, followed by two participants each with hypertension, diabetes mellitus, and panic disorder, and one with chronic lower back pain. The detailed demographics are summarized in Table 1.

The following section reflected on emergent themes and sub-themes in the current research. Four major themes and eleven sub-themes identified. 

**Figure 1:**
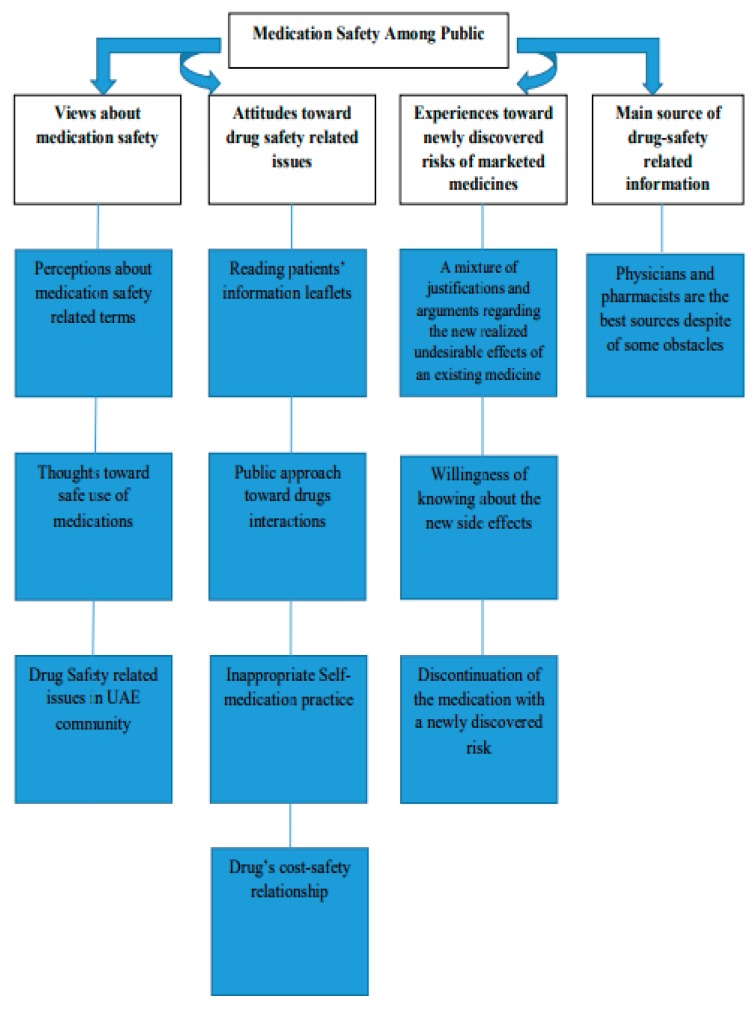
Emergent themes and sub-themes.

The main themes were views about medication safety, attitudes toward drug-safety-related issues, experiences toward newly discovered risks of marketed medicines, and main source of drug-safety-related information. Figure 1 represents the emergent themes and sub-themes.

### 3.2. First Theme: Views about Medication Safety

#### 3.2.1. Sub-Theme 1: Perceptions about Safety-Related Medication Terms

Some participants have shown inappropriate understanding of the following terms: 

##### Effective Drug

- **P13**. The effective medicine works very fast with all of the patients with the same conditions.

##### Safe Drug

One participant was incorrectly able to distinguish between effective medication and safe medication.

- **P2.** Effectiveness of the medicine means that this medicine is giving excellent effect without causing any side effects.

Few participants have explained the meaning of safe drugs as they are not causing any harm if they have been consumed in amounts which are higher than their specified doses.

- **P5.** Safe drug means it will not cause any negative effects upon exceeding the dose which is mentioned in the leaflet.

##### Side Effects and Adverse Drug Reactions:

It has been found that all the participants understood that the terms of side effects and adverse drug reactions are equivalent in their meanings and they can be used simultaneously.

- **P1:** The drug that has no side effects has no adverse drug reactions. There is no difference between them.
- **P2:** Adverse drug reactions mean that the drug has no side effects.

Additionally, all the participants have mentioned drug interactions as an example of the side effects of the medications.

#### 3.2.2. Sub-Theme 2: Thoughts toward Safe Use of Medications

When the participants were asked in the interview about what questions they would ask their physician when prescribing a new medicine, most of the participants answered that they were viewing medications side effect as a major concept while consuming medicines, and all of them agreed that they would ask the physician about the side effects that may result upon using the prescribed new medication.

- **P5**: Definitely I will ask about the side effects that this new medication will cause.
- **P6**: The most important thing to ask about is the possible side effects which may appear after having this newly prescribed medication.

All the participants with the chronic diseases have shown a high interest in asking the physician about the drug–drug interactions of the newly prescribed medication to ensure that it will cause no harm while consuming it with their regular medicines. However, the drug–drug interactions related aspect has not been mentioned by the rest of the participants. 

Moreover, it has been revealed that few participants have considered the indication and dosage of the prescribed/dispensed medication as important aspects that the patient has to confirm before consuming the newly prescribed medications.

- **P10**: I am sure that the main points I would ask about are: all the indications of this medicine, its dosage and side effects.
- **P11**: It is very necessary to ask about the dose of any newly prescribed medicine. Plus, I would like to reconfirm that it suits my condition since it is the first time for me to use it.

#### 3.2.3. Sub-Theme 3: Drug-Safety-Related Issues in the UAE Community

Participants were asked about important drug safety problems encountered in the UAE community, from their points of view. Only, two participants stated that there were no problems regarding this in the UAE, while the rest of the participants mentioned at least one of the following problems:

Antibiotics are prescribed excessively by physicians and pharmacists. Many participants agreed that once the physician or pharmacist finds that the temperature of the patient is raised, he/she will prescribe an antibiotic. 

The participants argued that viral infections are not ruled out by the physicians before prescribing the antibiotics.

- **P11**: Physicians usually prescribe antibiotics for all the patients who are suffering from high temperature.

Participants mentioned that the reasons for prescribing antibiotics were not always clarified to the patients.

- **P12:** One time a pharmacist had prescribed me an antibiotic when I complained of feeling dizziness.

Many participants have recognized that physicians are not adherent to the medicines’ doses which are specified in the patient information leaflet and more specifically for pediatric patients.

- **P11**: Most of the time, when coming back home from the clinic and after referring to the medicine leaflet, I find that the prescribed dose is different.
- **P4**: Always the doses of the medicines which were prescribed for my children are lower than what has been mentioned in medicine’s brochure.

All the medications were dispensed without considering the other medications which were taken by the patients. All the participants who were suffering from chronic diseases have negotiated this issue. A participant who had chronic diseases and especially who had multiple conditions were worried to take any medicine without knowing its related drug–drug interactions. They suggested that patients have to be asked about all their consumed drugs and all the related drugs interactions have to be mentioned to the patients by physicians and pharmacists too.

- **P2***:* Without asking the physician or the pharmacist nobody is asking me whether I take other medications.
- **P14***:* No healthcare professional is making sure that my prescribed medicine is contraindicated with my chronic medicines or not.

### 3.3. Second Theme: Attitudes toward Drug-Safety-Related Issues 

#### 3.3.1. Sub-Theme 1: Reading Patients’ Information Leaflets

It was realized that most of the participants would refer to the patient information leaflets included in the medications that they (their children, or relatives) would use. There are three main reasons why participants engaged in this.

They have stated that gaining knowledge about the side effects of the prescribed drug was the main reason for reading the patient information leaflets. In this regard, participants have addressed the importance of knowing all the side effects of the consumed medications. They agreed to be familiar with the possible side effects of the medicine rather than guessing the side effects, and possibly provoking a new disease without knowing that the taken medication was the contributing factor.

- **P9**: I have the right to know all the side effects of the used medication; therefore, I refer to the product’s inserted leaflet.
- **P7**: I am not satisfied with the explanation of the healthcare personnel regarding the side effects of my medications. That’s why I usually read them in details from the leaflet.
- **P8**: I always like to read the medicine leaflet and ask the pharmacists about my suspicions before starting the medications.

Confirming that the prescribed dosages were correct was considered another reason for reading the patients’ information leaflet. Many participants raised the issue of prescribed drugs with incorrect doses. Not all the participants trusted the doses that were set by the physicians. This was based on previous experiences where the doses were found to be inappropriate. It was more commonly stated by the mothers of children.

- **P4:** I always find that the prescribed doses are incorrect for my child’s age and weight.
- **P11**: I don’t trust the dose mentioned by the physician or the pharmacist. I always check the dosage from the medicine’s leaflet.

One more reason that was leading the participants to read the patients’ information leaflets was being aware of the medication contraindications. Many participants and especially those with chronic diseases were keen about knowing the conditions in which the currently prescribed drug was contraindicated.

- **P1**: I am a diabetic patient, and I must read before using any drug whether it will affect my blood sugar level or not.
- **P2**: I must ensure that the newly initiated medication is not contraindicated with my hypertension.

#### 3.3.2. Sub-Theme 2: A Public Approach toward Drugs Interactions:

It was recognized that except for chronic users of medications, insufficient attention regarding drugs interactions and their complications were directed toward the participants’ consumed medications.

- **P10**: No healthcare provider gives me importance regarding information of drug interactions, therefore I think it something rare that happens and I don’t think about it.

During the interview when the issue of drugs interactions was discussed, many participants were found to be underestimating the seriousness of drugs interactions.

- **P5**: I don’t think that drug interactions will cause severe harm or death to any patient.

Ignorance of drugs interactions related issues were observed with many participants.

- **P6**: I don’t care about it. If any drug interaction is found to be serious, definitely it will be mentioned by a physician.

#### 3.3.3. Sub-Theme 3: Inappropriate Self-Medication Practice 

During the interview, participants were asked whether they used a medication for an indication other than the indication for which it was prescribed. The self-medication practice issue has evolved. It was found that self-medicating attitudes became a common practice in the UAE. In this regard, many participants mentioned that pharmacist’s consultations were not required for the use of herbal products and supplements, as they are completely safe and show no side effects.

- **P12**: I don’t ask the pharmacist or even the doctor if I decide to take vitamins and herbal products because they are safe.
- **P5**: Herbs are not harmful products even if the person takes them at high doses.

On the other hand, some participants disagreed completely with consuming any herbal or supplement without referring to healthcare providers, since they believed that the inappropriate use of such products might negatively affect their conditions.

- **P1**: Definitely, I have to seek my doctor’s advice before taking any supplements as it may be contraindicated with my condition. For example, I take aspirin to increase the fluidity of blood, and I know that garlic containing products may increase the possibility of bleeding in such situation. Therefore, I have to ask before taking any product.

Moreover, some participants mentioned that they commonly use certain medications according to their previous related self-experiences.

- **P6**: I have repeated the course of antibiotics based on what I have been prescribed in the past when I develop a similar condition.
- **P4**: As far as I remember, I had a cough and the pharmacist gave me medicine. Later, when I got a cough again, I have used that medicine without asking anybody.
- **P10**: Sometimes I read the leaflet and I find many uses for the medication that I take. In the future when I develop any of these conditions or diseases that are listed in the leaflet, I don’t mind to use the medication. But I don’t do this with my friends and relatives.

All the participants have stated that friends and relatives’ suggestions of medications are not trustable completely. Two participants shared their own stories on how taking a medication that was suggested by their friends and families had resulted in a bad experience.

- **P7**: One of my friends recommended me a natural product. I took it, and a few days later I had very high and disturbing palpitations. I was scared and asked the pharmacist who clarified to me that it was a side effect of the product. I stopped it immediately and decided never to trust any friend’s suggestions.
- **P9**: I usually suffer from insomnia. I was suggested to take Phenergan tablets 2 hours before my sleeping time. The next day I fainted down in the street and when the first aid people arrived, they informed me that I suffered from severe hypotension. Therefore, I stopped believing in the friends’ suggestions for medications.

#### 3.3.4. Sub-Theme 4: Drug’s Cost–Safety Relationship

It was revealed that there was a common belief among the participants that costly drugs are of a high-safety profile. Most of the participants perceived that expensive brand medication is more effective and safer than the cheap ones. Moreover, most of the participants have shown a high tendency to buy expensive medications.

- **P1**: I always prefer to get an expensive form of the medication because I think it is stronger and causes fewer side effects.
- **P8**: The expensive medicine is much better than the cheap one. Because I think it is highly pure medicine, therefore, it will not cause serious side effects.

### 3.4. Third Theme: Experiences toward Newly Discovered Risks of Marketed Medicines

#### 3.4.1. Sub-Theme 1: A Mixture of Justifications and Arguments Regarding the Newly Realized Undesirable Effects of an Existing Medicine.

The participants were asked the following question: “Sometimes, even after prescribing medicines which are available for a long time in the market, the public will hear about newly discovered risks of using the drug. Why do you think this kind of thing happens?” Their replies were summarized in the following two statements:

- Some participants have justified the newly discovered risks due to the difficulty of gathering the entire drug-related risks (by the pharmaceutical companies) before the marketing stage.
- On the other hand, some other participants argued and related such newly discovered risks of already marketed products due to the shortage of the clinical trial period of the concerned drug. They said that this was the main cause of newly appearing side effects post-marketing.

#### 3.4.2. Sub-Theme 2: Willingness of Knowing about the New Side Effects

Additionally, they showed their willingness of knowing about the new side effects. However, most of them stated that the depth of reading and being aware of the new side effects was limited to the concerned consumed medicine. They were not interested in knowing about such topics in general.

#### 3.4.3. Sub-Theme 3: Discontinuation of the Medication with a Newly Discovered Risk

Upon asking the participants about their reactions toward the newly discovered risk of the medications in use, all participants without exception mentioned they would discontinue the medications in the case that new risks or undesirable effects were observed or discovered while they were consuming these medications, regardless of the indications. Looking for alternative medications was the agreed solution among all participants.

- **P7**: I will stop it immediately and consult the doctor for the alternative one.
- **P8**: I am not going to continue a medication with side effects even if I don’t develop those side effects.
- **P2**: I will not trust this medication anymore; I will not use it at all. I will consult the physician to find the alternative.

### 3.5. Fourth Theme: Main Source of Drug-Safety-Related Information

#### Sub-Theme: Physicians and Pharmacists Are the Best Sources Despite Some Obstacles 

Surprisingly, most of the participants believed and referred to pharmacists as one of the main sources of drug-related information, specifically, if they have certain drug-safety-related issues. They justify their attitudes by the easiness of access to pharmacies, and they are highly aware that pharmacists are knowledgeable in the field of drugs information. At the same time, they did not neglect the role of physicians in such domain. However, some obstacles in this regard have been mentioned by the participants. 

Many participants observed and criticized the lack of proper communication between pharmacists and physicians and they viewed that it is a major issue where the pharmacists sometimes are not able to tell the drug-safety-related information which is suitable for the patient because of an insufficient interaction with a physician regarding the patient’s case.

- **P9**: I feel myself confused when there is no communication between the pharmacist and the physician that I consulted, especially when I visit more than one physician.

## 4. Discussion 

The purpose of the current research was to explore the general public views, attitudes, and experiences towards medication safety topics, their concerns, and gaps identified in this area. The findings have shown that the general public have appropriate knowledge and positive attitudes about the safe use of medications; however, they were unaware of the following points: accurate definitions of some related terms like drug effectiveness and side effects, the concept of drug interactions, the physicians prescribing patterns of drugs, e.g., antibiotics, antihistamines. Moreover, they have reported their incomplete satisfaction regarding medication safety-related information which is provided by healthcare providers. Inappropriate self-medication practice and a misbelief that costly medications are the safest was also identified. Concerning the post-marketing of adverse drug reactions (ADRs), none of the participants mentioned ADR reporting actions, although they justified confidently the underlying background of this issue. Additionally, the shortage in physician–pharmacist relationships was unsatisfying many participants toward ensuring their medications’ safety. Most of the participants showed appropriate views and perceptions toward medication safety in general which is in accordance with the previous study [14], whereas other findings have reported a low-level of medication safety knowledge among participants in other countries [17,27,28].

In terms of perceptions toward definitions of some related terms like drug effectiveness and side effects, the majority stated the definitions of some related terms. for example, effective drug, side effect, and ADRs correctly and appropriately, but misapprehensions in some answers have also been observed as few mentioned the definitions wrongly and considered themselves right. This is in line with the findings of Hassali et al. 2012 [27]. 

Regarding the thoughts towards safe use of medications, drug interactions are one of the major safety aspects of medicine use that is important for patients to know about [17,29]. In this regard it was revealed that participants with chronic diseases were used to focusing on the drug interactions through the interviews. On the other hand, healthy participants did not confer about drug interactions at all and the risks of drug interactions have been underestimated by them. This result can be justified as usually patients with chronic diseases use multiple medications regularly and therefore, are at a higher risk of developing drug interactions [8]; however, individuals should be aware of this issue to enhance the medication safety. A study [30] that was conducted in Ajman, UAE has concluded that intervention is required to improve peoples’ knowledge about drug interactions to develop medication safety. This conclusion was supported by an American study which indicated the importance of drug interaction related knowledge among patients [15].

For drug safety issues in the UAE community, many participants reflected that they could not understand their physicians’ pattern of prescribing medicines for their illnesses where most of them have focused on antibiotics as an example. This result found to be parallel with the results of another study which has been conducted in the UAE in 2010, and which concluded that 85% of the patients requested to confirm the appropriateness of the prescribed medications to them [31]. Specifically, the given antibiotic as an example was due to the problem of overusing of antibiotics in physicians’ prescriptions, and this result is in line with the results of other countries in the middle east [31,32,33]. 

Many participants reported their incomplete satisfaction regarding medication-related information provided by healthcare providers (physicians and pharmacists). The concomitant administration, indications, doses, side effects, and contraindications of the prescribed drugs were used as examples by the participants in this study. This outcome was supported through evidences from Australia [34], USA [35], Canada [36], Malaysia [27], and Oman [37]. Evidence suggested the importance of patient involvement in therapeutic decisions and to involve the patient as a “vigilant partner” to ensure medication safety [38]. Insufficient information exchange between HCP and patients may negatively impact patients’ safety [39], and this will not be achieved unless HCP would implement patient-centered care approach to meet medications consumers’ needs [40].

In the concept of inappropriate self-medication practice, the participants revealed that self-medication is a common practice among the lay public which was also reported from one study from Denmark [41]. Participants declared that pharmacists consultations were not required when using herbal products and supplements, as they are completely safe. On the other hand, participants also disagreed completely to consume any herbs or supplements without referring to a health care provider, since they believed that the inappropriate use of such products might negatively affect their conditions. Moreover, some participants mentioned that they commonly use certain medications according to their previous related self-experiences. In this regard, antibiotics have been stated as an example by the participants frequently. Self-medication is defined as the selection and use of medicines by individuals (or a member of the individuals’ family) to treat self-recognized or self-diagnosed conditions or symptoms [42]. However, self-medication (especially the irresponsible medication use) has potential risks, e.g., severe adverse reactions, dangerous drug interactions, incorrect manner of administration, incorrect dosage, incorrect choice of therapy, and masking of a severe disease [42], which can be considered as a drug-related problem [41]. Vickers and Zollman commented that herbal medicine most likely poses a great risk of adverse effects and interactions. There were case reports of serious adverse events after administration of herbal products in which the herbs involved were self-prescribed and bought over the counter or obtained from a source other than an HCP [43]. Self-medication with antibiotics has been reported locally in the UAE [44,45] and globally all over the world. However, over the counter sales of antibiotics is expected to be overcome currently by issuing laws against buying antibiotics without prescriptions. The UAE Health Ministry will issue new health legislation to put an end to the dangerous practice of dispensing antibiotics without medical prescription [46]. 

Regarding participants’ belief of drug’s cost-safety relationship, there was a misbelief that costly medications are the most effective and safest. Previous research studies from Saudi Arabia [47] and Auckland [48] have reported similar results. Conversely, there is a study which reported that patients agreed on the cost-effective nature of generics with good quality than brand-name counterparts, and are equally safe [49]. Once cannot deny that literature also reported about consumers’ beliefs about medication cost issues [50]. 

In terms of experiences towards post-marketing adverse drug reactions, the current research did not highlight of any participant’s ADR reporting action; although they could justify confidently the underlying background of this issue. Moreover, they have shown their willingness to know about the new side effects of their own medicines. Lack of knowledge about ADR reporting to the national PV centers among the public has been observed in many relevant studies. Jimmy et al. 2015 [37], Matos et al. 2015 [51], and Elkami et al. 2013 [16] can be cited as supporting evidence in this regard. However, the public has shown their interest to report ADRs [52]. Two systematic reviews [53,54] have concluded to encourage the involvement of the patients in ADR reporting systems and thus to enhance patient safety. 

For the sources of drug-safety-related information and the corresponding obstacles additionally, many participants have realized a shortage in physicians–pharmacists relationships which were frustrating them toward ensuring their medication safety. It is highly important to consider this collaborative working relationship (CWR) as it fosters the provision of pharmaceutical and patient care activities, and thus enhances patient safety [55,56].

Successful HCP CWR was found to improve patient safety [56]. Developing collaborative working relationships between physicians and pharmacists can assist healthcare practitioners in developing a team-based approach to patient care, improving the ability of pharmacists and physicians to work together to coordinate patient care [57].

In the current research, participants have agreed that physicians and pharmacists were the best sources of drug information and especially if drug safety concerns were existing. Previous research from Saudi Arabia [47], Oman [37] and Malaysia [5] reported similar findings and already cited by Nair et al. that pharmacists are the most accessible source of information for individuals because they are naturally available when patients needed information [36].

The current research attempted to control researcher bias and the principal researcher maintained a reflective diary and penned down reflections and thoughts after the commencement of every interview. 

## 5. Conclusions

In conclusion, this study has revealed a gap in the public views regarding the medication’s safety, which consequently may impact their attitudes while using their medications and in turn affect their safety. The current research was found to underestimate the risks that would be evolved from drug interactions and self-administration of supplements and vitamins. Participants lacked inspiration of discussing to their HCPs about their medicines’ side effects, and also possessed no background of the ADR reporting system in UAE. Therefore, interventions should be established to improve public perceptions toward medication safety and to increase their awareness about the importance of being a part in the ADR reporting system. Self-medication has to be guided by the HCP, and community pharmacists should play an active role in educating and counseling of medications. Furthermore, individuals have the right to clarify their doubts regarding their own medications and accordingly opportunities of effective communication with pharmacists have to be encouraged. Public need to undergo professional supervised educational programs to establish strong background of medications safety among the consumers. Further research evidence is required to reveal the most suitable means of improving medications safety among public. 

## 6. Limitations

Participants took lot of time to recall some relevant stories from their own experiences regarding the topic of the interview, and thus in such cases it was preferred that the list of questions must be provided before the interview for preparing the answers beforehand. The transferability of the findings poses limitations as the data was able to explore the views of only a couple of locals and mostly comprised of non-locals. Secondly, the current research is a part of mixed-method research and it is anticipated that experiences towards the newly discovered risks of marketed medicines might not be addressed. 

## Figures and Tables

**Table 1:** Patients’ demographic characteristics.

Total Number of Participants	14 (P1–P14)
Age	22–64 years old
**Gender**	
Male	6
Female	8
**Nationality**	
Local	2
Non-Local	12
**Education Level**	
Secondary School	5
University	9
**Health Status**	
Healthy	7
Chronic Diseases	7
